# Application of Bioelectrical Impedance Analysis in Nutritional Management of Patients with Chronic Kidney Disease

**DOI:** 10.3390/nu15183941

**Published:** 2023-09-12

**Authors:** Yanchao Guo, Meng Zhang, Ting Ye, Zhixiang Wang, Ying Yao

**Affiliations:** 1 Department of Nutrition, Tongji Hospital, Tongji Medical College, Huazhong University of Science and Technology, Wuhan 430030, China; ycguo@tjh.tjmu.edu.cn (Y.G.); yeting@tjh.tjmu.edu.cn (T.Y.); 2 Department of Nephrology, Tongji Hospital, Tongji Medical College, Huazhong University of Science and Technology, Wuhan 430030, China; zegang1988@163.com (M.Z.); tongji-wzx@tjh.tjmu.edu.cn (Z.W.)

**Keywords:** body composition, bioelectrical impedance analysis, chronic kidney disease, nutrition

## Abstract

Body composition measurement plays an important role in the nutritional diagnosis and treatment of diseases. In the past 30 years, the detection of body composition based on bioelectrical impedance analysis (BIA) has been widely used and explored in a variety of diseases. With the development of technology, bioelectrical impedance analysis has gradually developed from single-frequency BIA (SF-BIA) to multi-frequency BIA (multi-frequency BIA, MF-BIA) and over a range of frequencies (bioimpedance spectroscopy, BIS). As the clinical significance of nutrition management in chronic kidney disease has gradually become prominent, body composition measurement by BIA has been favored by nephrologists and nutritionists. In the past 20 years, there have been many studies on the application of BIA in patients with CKD. This review describes and summarizes the latest research results of BIA in nutritional management of patients with CKD including pre-dialysis, hemodialysis, peritoneal dialysis and kidney transplantation, in order to provide reference for the application and research of BIA in nutritional management of chronic kidney disease in the future.

## 1. Introduction

Body composition measurement plays an important auxiliary role in nutritional assessment, nutritional diagnosis, and evaluation of nutritional therapy in a variety of diseases. Alterations in body composition, such as wasting and stunting, can be observed when nutrient intake is inadequate. On the other hand, overnutrition can lead to obesity. In terms of nutrition, body composition is mainly divided into water, muscle, fat, and inorganic salts. Many techniques and methods exist for body composition determination, ranging from simple indirect measurements to complex direct volumetric measurements [1]. At present, the detection methods of body composition that are well known and used for research mainly include anthropometry, tracer dilution method, densitometry, dual-energy X-ray absorptiometry (DEXA), air displacement plethysmography, magnetic resonance imaging (MRI), computed tomography (CT), and bioelectrical impedance analysis (BIA) [2]. At present, DEXA is considered as the gold standard for body composition measurement. The principles of the above-mentioned human body composition detection methods are different, and their precision, accuracy, radiation exposure, convenience and cost are also different, and they have their own advantages and disadvantages. There has been relevant review for a detailed summary [3], so this article will not repeat this part of the content.

BIA first appeared as a method for the analysis of body composition following the publication of Lukaski and colleagues’ seminal paper in 1985 [4]. In the past 30 years, BIA has been widely used and studied in many disease fields, including nutritional management (sarcopenia, obesity, malnutrition, etc.) and disease nutrition (heart, liver, kidney, tumor, etc.) [5]. The main advantages of BIA are its non-invasiveness, no radiation, economy, convenience and feasibility [3]. The basic principle of BIA is to take the human body as a conductive cylinder, use the electrical properties of the inner and outer fluid and the cell membrane of the human body, measure the resistance (R) and reactance (X_C_) at different electrical frequencies, and then estimate the body composition by empirical regression equation. These include intracellular and extracellular water content, fat-free mass, muscle, fat, inorganic salts, body cell mass, phase angle, etc. The electrical resistance (R) is mainly determined by the electrical properties of the intracellular and extracellular fluids, and the intracellular and extracellular water content can be calculated, respectively. The reactance (X_C_) is mainly determined by the capacitive properties of the cell membrane and reflects the somatic cell population. Phase angle (PhA) is an indicator of the relationship between X_C_ and R, which can reflect the integrity of cell membrane structure and function [6].

With the development of bioelectrical impedance technology, BIA has gradually developed from single-frequency BIA (SF-BIA) to dual-frequency BIA (DF-BIA). Currently, devices are available that measure at multiple fixed frequencies (multi-frequency BIA, MF-BIA) and over a range of frequencies (bioelectrical impedance spectroscopy, BIS). Different BIA instruments may use different correlation models and equations. MF-BIA and BIS mainly use high-frequency currents (>50 Hz) through the cell membrane to measure intracellular water, and low-frequency currents (≤50 Hz) mainly measure extracellular water. MF-BIA measures resistance at different frequencies and incorporates it into an empirical linear regression model to derive numerical values for body composition. Bioimpedance spectroscopy (BIS) uses mathematical modeling and mixed equations, such as the Cole–Cole formula and Hanai formula, to derive the relationship between resistance and fluid compartments to assess body composition [7]. MF-BIA techniques routinely express body composition as a two-compartment (2-C) model distinguishing fat mass (FM) and fat-free mass (FFM), which may be influenced by overhydration (OH). Approximately one decade ago, a three-compartment (3-C) model was introduced distinguishing OH, adipose tissue mass (ATM), and lean tissue mass (LTM) [8]. Both 2-C and 3-C models are currently used in clinical studies. Even though the accuracy of BIA body composition measurement is still questioned, it is still widely used in clinical and scientific research, and many studies have confirmed its important significance in nutritional management and disease prognosis [6].

In recent years, the clinical significance of nutrition management in chronic kidney disease (CKD) has gradually been highlighted [9]. Bioelectrical impedance analysis of body composition is favored by nephrologists and nutritionists as an auxiliary method for nutrition diagnosis, treatment and monitoring [10]. In the past 20 years, there have been many studies on the application of BIA in the nutritional management of CKD patients, mainly divided into several aspects: (1) comparison between BIA and other body composition measurements such as DEXA; (2) changes in body composition at different CKD stages; (3) implications of changes in body composition; and (4) the predictive power of related body composition changes for disease prognosis. The changes in body composition indicators detected by BIA in CKD patients were mainly concentrated in lean tissue, fat, phase angle, and body water. This article mainly elaborates and summarizes the latest research results of BIA in nutritional management of CKD patients with pre-dialysis, hemodialysis, peritoneal dialysis and kidney transplantation from the above four aspects.

## 2. Results

### 2.1. Compared with Other Body Composition Measurement

Dual energy X-ray absorptiometry (DEXA) is often used as the gold standard for body composition measurement [11]. In CKD patients, the agreement between DEXA measurement and BIA measurement is often used to evaluate the accuracy of BIA. Most studies believe that BIA has good consistency with DEXA in measuring fat and lean tissue in hemodialysis (HD) and peritoneal dialysis (PD) patients [12,13]. However, in terms of body cell number (BCM), some studies have suggested that the agreement between BCM measurements and DEXA is better in HD patients [14], but the agreement between BCM measurements and DEXA is poor in renal transplant patients [15]. Some studies have also suggested that the accuracy of BIA in measuring bone mineral content in HD patients is poor [16]. The reason for this may be the differences in BIA instruments involved in the studies, and the type and status of patients are also different. Therefore, more well-designed studies are needed to comprehensively evaluate the accuracy of BIA.

One study on fat-free mass (FFM) measured by BIA showed that FFM estimated by the Kyle, Sun SS and Segal equations was consistent with that measured by DEXA in healthy volunteers. However, for dialysis patients, FFM predictions of different equations differed between HD and PD patients, and these equations seemed to be more applicable to HD patients. The results showed that BIA equations based on healthy people may not be applicable to dialysis patients, especially PD patients [17]. One cross-sectional study involving both pre-dialysis CKD5 patients and dialysis patients suggested poor agreement between fat-free mass index (FFMI) measured by BIA and that measured by DEXA [18]. The above results suggest that specific BIA equations should be established for different types of CKD patients including non-dialysis and kidney transplant patients to obtain the most accurate results [15].

Some studies have used DEXA as the gold standard to compare the agreement between BIA and anthropometry with DEXA, suggesting that BIA is more suitable than anthropometry to measure skeletal muscle mass (SMM) [19] and fat mass (FM) [20] in HD patients. Another study compared the agreement between different BIA equations and DEXA in measuring fat percentage in HD patients and found that BIA–Kushner equation was more consistent than anthropometric and BIA–Segal/Lukaski equation methods [21]. However, some studies have suggested that in non-dialysis CKD or HD patients, the consistency of BIA and DEXA is similar [22] or even worse than anthropometry [23,24,25]. A systematic review of similar studies is warranted, but more research is needed to draw reliable conclusions.

In addition, for patients with the same type of CKD (pre-dialysis/HD/PD/renal transplant), patients with overhydration were less accurate in fat and muscle measured by BIA than patients with normal hydration [26]. Body fat percentage measured by BIA in HD patients with less than 34.4% body fat percentage was less accurate than those with more than 34.4% body fat percentage [27]. This suggests that for the same type of CKD patients, hydration status and percentage body fat content may affect the accuracy of body composition by BIA.

### 2.2. Pre-Dialysis CKD

#### 2.2.1. Lean Tissue and Fat

The percentage of muscle mass (MM%) of pre-dialysis-CKD4-5 patients is generally lower than that of healthy controls [28]. In patients with pre-dialysis CKD3, for every 10 mL/min/1.73 m^2^ decrease in glomerular filtration rate (GFR), whole-body muscle mass (MM) decreases by 0.59 kg [29]. The skeletal muscle mass index (SMI) of CKD5 patients before dialysis was significantly lower than that of CKD1-4 patients [30].

A low protein intake was associated with loss of MM in the CKD3-5 patients [31]. One study found that upper-limb fat-free mass (FFM) measured by BIA was positively correlated with grip strength, whereas fat mass was negatively correlated with grip strength [32]. Pre-dialysis CKD5 patients with higher lean tissue index (LTI) have higher quality of life [33]. Among pre-dialysis CKD patients, men had a higher MM% and women had a higher percentage of fat mass (FM%) [34]. FFM measured by BIA performed best in the diagnosis of low muscle mass, while fat mass index (FMI) and waist-to-hip ratio (W/H) were the best parameters for the diagnosis of obesity [35]. In one study, FM% measured by BIA was used as the diagnostic index of obesity, and the results of 30 years of follow-up showed that obesity was a risk factor for renal function decline and renal function attenuation [36].

One study showed that the incidence of sarcopenia in pre-dialysis CKD patients was 24%. The sarcopenic individuals had lower eGFR, BMI, fat tissue index (FTI) and higher prevalence of PEW [37]. Sarcopenic obesity was prevalent in 62.5% of the pre-dialysis CKD patients. In subjects with low muscle mass, a decrease in eGFR is significantly associated with a decrease in body weight, body fat mass and visceral fat area [38]. In one study, skeletal muscle mass index (SMI) measured by BIA was used to determine sarcopenia compared with anthropometric measurements or subjective global assessment (SGA), and only sarcopenia diagnosed by SMI remained as a predictor of mortality after multivariate adjustment [39].

Protein-energy wasting (PEW) is associated with increased mortality, and the prevalence in non-dialysis patients ranges from 0 to 40.8% [34]. Pre-dialysis CKD patients with PEW had lower FM and MM [34]. One study suggested a significant inverse association between LTI and incidence of malnutrition in patients with diabetic chronic kidney disease stage 5 (DMCKD5) [40].

#### 2.2.2. Phase Angle

The phase angle (PhA) of patients with advanced pre-dialysis CKD is lower than that of healthy people [41]. A decrease in phase angle was significantly associated with a decrease in estimated glomerular filtration rate (eGFR) [38]. The PhA in the diabetic chronic kidney disease stage 5 (DMCKD5) group was significantly lower than that in non-diabetic CKD stage 5 (nDMCKD5) group. PhA was positively correlated with lean tissue index (LTI), albumin and hemoglobin levels [40]. PA was positively correlated with MM% and BCM%, and negatively correlated with high-sensitivity C-reactive protein (Hs-CRP) [28]. The level of phase angle was lower in the lowest protein intake quartile than in the highest protein intake quartile [42].

An observational study suggested that the PhA could be used as a marker to reflect the nutritional status in patients with DMCKD5 [40]. One study directly defined PhA less than 4.5° as PEW. The PhA was positively correlated with geriatric nutritional risk index, lean mass index (LTI) and albumin, and negatively correlated with the ratio of overhydration to extracellular water (OH/ECW) [43]. In a retrospective cross-sectional observational study of CKD stage 3 (CKD3) patients followed for 10 years, survivors had significantly higher body cell mass (BCM%) and PhA levels. Survival analyses significantly showed that age > 72 years PhA ≤ 4° were associated with an increased risk of mortality [44]. In a multicenter cohort study involving 427 pre-dialysis patients (mean GFR, 7.0 ± 8.7 mL/min/1.73 m^2^), the PhA decreased by a mean of 0.6 degrees as patients transitioned to dialysis, but this change was not associated with the risk of heart failure, stroke, myocardial infarction, or all-cause mortality [45]. Thus, more studies are needed to confirm the predictive power of PhA for clinical outcomes when disease stages change in CKD patients.

#### 2.2.3. Body Water

One study showed that 40% of CKD3 patients had fluid overload before dialysis [46]. Another study showed that 62% of CKD3b patients with stable renal function had clinically unrecognized fluid retention following 9 months of judicious dietary protein intake (0.6–0.8 g/kg/day) [47]. It also showed that total body water was slightly higher (+4.3% in men; +3.5% in women) in 84 CKD4-5 patients compared with 604 healthy control subjects [41]. An observational study suggested that the pre-dialysis DMCKD5 group had more severe overhydration (OH) than the nDMCKD5 group [40]. Another research showed that the incidence of OH in CKD5 patients before dialysis was higher than that in PD patients [43]. It was found that the ratio of extracellular water to total body water (ECW/TBW) in CKD3-5 patients was associated with decreased intracellular water (ICW) and weight loss due to malnutrition or aging [48].

### 2.3. Hemodialysis

#### 2.3.1. Lean Tissue and Fat

One study showed that the lean body mass (LBM) and body cell mass (BCM) were all significantly decreased after hemodialysis [49]. Another research showed that the lean body mass index (LTI) value was significantly higher in male patients than in female patients [50]. Two studies confirmed that fat free mass (FFM) of HD patients with overweight or obesity was higher than that of patients with normal weight [51,52]. The LTI of HD patients was positively correlated with serum albumin [50,53] and serum prealbumin [50]. The LTI was also positively correlated with protein intake [53,54] and body cell mass (BCM) [53], but negatively correlated with age [53]. Another study of 52 HD patients showed that percentage of muscle mass (MM/%) was independently inversely associated with inflammation [55]. A study involving 155 HD patients showed a positive correlation between LTI and grip strength (HGS) in both men and women [53]. Another study of 97 HD patients revealed that the muscle quality index (MQI, the ratio of grip strength to arm muscle mass measured by BIA) was associated with gait speed [56].The percentage of body cell mass (BCM%) was found to be inversely correlated with age. Patients with diabetes had lower BCM% than those without diabetes in two observational studies [57,58]. In a study of 48 HD patients, it was observed that BCM was positively correlated with lean tissue mass (LTM) and HGS, and negatively correlated with IL-6 concentration [59]. A multi-center study involving 774 HD patients showed that BCM was the determinant of resting metabolic rate (RMR) measured by BIA in multiple linear regression models [60]. The results of two other studies showed that LTM or muscle mass (MM) was the only significant determinant of resting energy expenditure (REE) measured by indirect calorimetry in HD patients [61,62].

In a cohort of 8227 patients, BMI increased about 0.6 kg/m^2^ over 24 months from baseline (6 months after renal replacement therapy initiation). This was associated with an increase in fat tissue index (FTI) of approximately 0.95 kg/m^2^ and a decrease in lean tissue index (LTI) of approximately 0.4 kg/m^2^. Female gender, diabetes status, and low baseline FTI were associated with significantly greater increase in FTI. Age > 67 years, diabetes, male gender, high baseline LTI, and low baseline FTI were associated with a significant greater decline of LTI [63]. In a study of 149 HD patients, percentage body fat (BF%) < 15% significantly predicted higher mortality [57]. In a prospective cohort study of 375 HD patients from seven hospital dialysis centers, the results suggested that the higher values of body fat mass (FM) and BF% were associated with lower mortality [64]. In a multicenter cross-sectional study of 609 HD patients, waist circumference and BF% estimated by BIA were used as proxies for visceral and subcutaneous fat, respectively. Surrogate measures of visceral and subcutaneous fat appear to have opposite associations with biomarkers of inflammation and nutrition. Subcutaneous fat may be an indicator of nutritional status, whereas visceral fat may be an indicator of inflammation [65]. A study of 188 HD patients found that for every one unit increase in visceral adiposity index (VAI) measured by BIA, the Framingham risk score, which assesses cardiometabolic risk factors, increased by 1468 units, indicating that excess visceral fat area increases the risk of cardiovascular disease [66].

A study involving 100 HD patients observed that patients with combined protein energy wasting (PEW) had lower fat mass, fat ratio, visceral fat mass, lean body mass, and muscle mass compared with well-nourished patients, as determined by BIA findings [67]. A multicenter cohort study of 5422 HD patients showed that PEW patients presented higher age, fat tissue index (FTI); lower dry weight, BMI, body cell mass index (BCMI), and lean tissue index (LTI). In a multivariate Cox regression analysis, the combination of normalized protein catabolic rate (nPCR) < 1.0 g/Kg/day, albumin < 3.8 g/dL and BCMI < 6.4 Kg/m^2^ was a strong independent predictor of mortality in these patients (HR: 1.48) [68]. Conversely, in another study of 91 HD patients, undernutrition detected by BCM decision tree was an independent prognostic factor for PEW in multivariate analysis [69].

In one observational, longitudinal, multicenter study including 170 HD patients older than 60 years of age, patients in the sarcopenia combined with malnutrition group were older and had significantly lower SMI, BCM and BF. In survival analysis, the risk of death in the sarcopenia with malnutrition group was 2.99 times higher than that in the non-sarcopenia and non-malnutrition groups [70].

#### 2.3.2. Phase Angle

One study of 173 HD patients revealed that the phase angle (PhA) value was significantly lower than that of healthy controls (4.89° vs. 6.32°) [71], and the PhA value of HD patients with diabetes was significantly lower than that of HD patients without diabetes (5.5° vs. 6.9°) [57]. Relative to well-nourished patients, malnourished HD patients had lower PhA [72], and HD patients with sarcopenia had lower PhA relative to patients without sarcopenia [70]. In HD patients, PhA was higher in overweight or obese patients than in normal weight patients [51]. The results of both studies showed that PhA was a stronger predictor of PEW than BMI in HD patients with a cut-off value of 4.6° [71,73]. Another study also suggested that the lower PhA group was associated with a greater risk of PEW, with patients with PhA < 3.7° having a significantly higher risk of PEW than HD patients with PhA ≥ 5°. It has also been shown that PhA is the only variable that is independently and negatively associated with the risk of malnutrition in HD patients [74].

Another study involving 149 HD patients showed that PhA < 6° was significantly associated with reduced survival [57]. A study of 142 HD patients followed for an average of 29 months showed that a decrease in PhA was associated with an increased risk for death after adjustment for age, sex, and comorbidities. It was also found that PhA could predict the occurrence of infection, but was not associated with the occurrence of cardiovascular events [75]. However, a prospective cohort study of 194 HD patients with PhA ≤ 4.8° were 20 times more likely to die of cardiovascular complications compared with those with PhA ≥ 6.5° [76]. In another study, 250 HD patients were followed up for 2 years. HD patients with lower phase angles had lower grip strength and quality of life, higher rates of cardiovascular events, hospitalizations, and all-cause mortality [77]. One meta-analysis involved 32 observational cohorts revealed that the mortality risk of HD patients increased to 1.74 times with each one degree decrease in PhA [78].

#### 2.3.3. Body Water

One study suggested that the total body water (TBW), extracellular water (ECW) and intracellular water (ICW) were all significantly decreased after hemodialysis (HD). The TBW has the best agreement with clinical acute volume changes during HD compared with ECW or ICW changes alone [49]. In 155 patients treated with HD for ≥3 months, both men and women, overhydration (OH) was negatively correlated with grip strength (HGS). In men alone, overhydration was inversely associated with lean tissue mass (LTM) [53]. A study of 75 HD patients revealed that OH was associated with higher serum C-reactive protein (s-CRP) [79].

A multicenter cohort study of 5422 HD patients showed that when compared with the well-nourished patients, PEW patients presented relative overhydration assessed by BIA [68]. In a study of 158 HD patients, subjects with lower edema index had better nutrition according to subjective global assessment (SGA) (SGA A 0.391; SGA B 0.400; SGA C 0.413), and 17.72% of the patients died after a follow-up of 3.5 ± 1.15 years, who had significantly higher ECW/TBW values than the survivors (ECW/TBW, 0.408 vs. 0.393). The calculated ECW/TBW cut-off point for all-cause mortality was 0.4055, with sensitivity of 84.6%, and specificity of 69.8% [80]. A study of 90 HD patients concluded that the ratio of extracellular water to body cell mass (ECM/BCM) ≥ 1.20 was associated with high all-cause mortality and had a significant prognostic value for the risk of death in HD patients [81]. Another meta-analysis involving 32 observational cohorts showed that OH >15% predicted mortality in HD patients independent of the influence of comorbidity [78].

### 2.4. Peritoneal Dialysis

#### 2.4.1. Lean Tissue and Fat

Weight loss (LW) and weight gain (GW) were observed in peritoneal dialysis (PD) patients at 6 months after PD initiation. The LW group had a reduction in fat mass (FM) and body cell mass (BCM). In the GW group, FM and BCM increased [82]. One study followed up PD patients for 1 year, and found that in the GW group, the increase in FM was the most obvious, while all components in the LW group decreased simultaneously, including intracellular water (ICW), extracellular water (ECW), fat mass (FM), body protein volume (BPV), and bone mineral content (BMC) [83].

The prevalence of sarcopenia in PD patients was 11.5%. There was a significant correlation between the prevalence of sarcopenia and gender, with a higher incidence in male patients, which may be due to decreased secretion of testosterone-1 resulting in reduced muscle protein synthesis and increased muscle protein catabolism [84]. Higher lean mass index (LTI) is independently associated with higher peritoneal protein metabolism in PD patients, suggesting that better nutritional status plays a dominant role in peritoneal protein metabolism [85]. The cutoff value of LTI for predicting PEW was 12.95 kg/m^2^ [43].

The results of one study showed that the prevalence of obesity and sarcopenia obesity in PD patients was 11.4% and 3.8%, respectively. Percentage skeletal muscle mass (SMM%) was found to be negatively correlated with dyslipidemia measures, whereas percentage body mass (BF%) was positively correlated with these cardiovascular disease risk factors [86]. An observational study showed a baseline prevalence of sarcopenia of 13.8% in PD patients. After 2 years, 30.5% of the patients had a 10% decrease in lean tissue index (LTI) and 44.3% had a 10% increase in adipose tissue index (FTI). Longitudinal changes in LTI and FTI were more strongly associated with all-cause mortality than single values of LTI and FTI [87]. Visceral fat level was found to be an independent predictor of carotid-femoral pulse wave velocity (PWV) and brachial artery flow-mediated dilation (FMD) in PD patients, which can be used as one of the risk factors for cardiovascular disease in PD patients [88].

#### 2.4.2. Phase Angle

Six months after the start of peritoneal dialysis (PD), the patient’s phase angle (PhA) decreased significantly [82]. However, one study showed that PhA in the PD group was higher than that in the CKD5 group before dialysis [43]. The PhA of patients with protein energy wasting (PEW) was significantly smaller, and that a PhA less than 4.64° can well predict the occurrence of PEW [73].

The PhA was positively correlated with serum pre-albumin in PD patients. The cumulative survival rate of PD patients with PhA ≥ 6° was significantly higher than that of PD patients with PhA < 6°. PhA is an independent predictor of 2-year survival in PD patients [89]. One meta-analysis involving 32 observational cohorts revealed that the mortality risk increased to 1.74 times with each one degree decrease in PhA [78].

#### 2.4.3. Body Water

In PD patients, a decrease in the ratio of extracellular water to total body water (ECW/TBW) was associated with an improvement in nutritional status, while an increase in ECW/TBW was associated with the development of malnutrition [90]. Patients with fluid overload who undergo continuous ambulatory peritoneal dialysis have higher rates of peritonitis, cardiovascular events, and worse clinical outcomes than patients with normal hydration [91]. One study showed that the ECW/height ratio of PD patients correlated well with inflammation and echocardiographically-assessed volume burden [92]. The longer the duration of peritoneal dialysis, the greater the ratio of extracellular water to body cell mass (ECM/BCM), the higher the risk of death [93]. The ECM/BCM was a significant independent predictor of mortality (HR = 1.035) [94]. For every 10% increase in the ECM/BCM, the relative risk of death increased by about 35% [95]. However, another study showed that ECW/TBW as a continuous variable was not associated with an increased risk of death in PD patients [96]. It was shown that the ratio of overhydration to extracellular water (OH/ECW) was an independent predictor of mortality [96]. Another meta-analysis involving 32 observational cohorts revealed OH > 15% predicted mortality in PD patients independent of the influence of comorbidity [78].

### 2.5. Kidney Transplantation

#### 2.5.1. Lean Tissue and Fat

In the early stage of malnutrition after kidney transplantation (KT), the loss of muscle and fat can be screened by BIA monitoring [97]. Three months after KT, the muscle mass of the patients decreased significantly, and the percentage of body fat (FM%) was significantly higher than that before KT [98,99]. The skeletal muscle index (SMI) was 7.26 kg/m^2^ before operation, decreased to 7.01 kg/m^2^ at 1 month after operation, and increased to 7.55 kg/m^2^ at 12 months after operation. After adjustment, changes in SMI within 1 year were positively correlated with protein intake [100]. Body weight, BMI, fat-free mass (FFM), fat mass (FM), body cell number (BCM) and dry weight were significantly lower in the group with eGFR < 40 mL/min/1.73 m^2^ than in the group with eGFR > 40 mL/min/1.73 m^2^ after surgery. The FM% and visceral fat content of diabetic patients were higher than those of non-diabetic patients [101]. BIA was more sensitive to evaluate nutritional depletion than subjective global assessment (SGA) in transplant patients with borderline [102].

The Global Leadership Initiative on Malnutrition (GLIM) criteria were used to diagnose malnutrition in patients after kidney transplantation. Reduced muscle mass was assessed by MF-BIA and defined as an appendicular skeletal muscle mass index (ASMI) < 7 kg/m^2^ for men and <5.5 kg/m^2^ for women. According to GLIM criteria, 14% of kidney transplant recipients (KTR) were malnourished, of which 91% met the phenotypic criterion for reduced muscle mass. Similar results were found by using creatinine-height index (CHI, based on 24 h urine creatinine) as measure for muscle mass. This suggests that MF-BIA can be used as a tool to determine muscle mass in the diagnosis of malnutrition [103]. In another study, ASMI was used to diagnose sarcopenia in patients after kidney transplantation, and the incidence of sarcopenia was 18.4% [104].

#### 2.5.2. Phase Angle

Kidney transplant recipients (KTR) had a lower PhA than healthy individuals [105]. The PhA was lower in diabetic KTR than in non-diabetic KTR [101]. PhA and BMI were negatively correlated with sarcopenia after adjusting for age, sex, dialysis vintage, time after transplant, presence of diabetes mellitus, hemoglobin, eGFR, and other nutritional factors. The results suggested that renal transplant recipients with low PhA and BMI have a higher risk of sarcopenia [106]. One study showed that low PhA at discharge was associated with increased protein-energy wasting (PEW), as indicated by lower concentrations of nutritional biomarkers (plasma albumin, hemoglobin) and an active inflammatory response (ferritin), whereas in women, higher PhA before renal transplantation predicted greater grip strength 6 months after renal transplantation [99]. An 8-year follow-up study of patients after KT showed that compared with a PhA greater than 5.85, a phase angle value lower than 5.85 indicated a 5.33 times higher risk of mortality [107].

#### 2.5.3. Body Water

It has been shown that the ratio of extracellular water to total body water (ECW/TBW) was higher and ratio of intracellular water to total body water (ICW/TBW) was lower in KTR than in healthy controls [105,108]. The ECW/TBW was higher and ICW/TBW was lower in the eGFR < 60 mL/min/1.73 m^2^ group, compared with the eGFR ≥ 65 mL/min/1.73 m^2^ group after kidney transplant operation [109]. The KTR with ECW/TBW > 0.40 had a higher incidence of cardiovascular disease than those with ECW/TBW ≤ 0.40 [110]. The total body water (TBW) content was higher in KTR who took more than one antihypertensive drug than in those who took only one drug. It suggests that there is an association between hypertension and overhydration in KTR. It suggests that KTR with high edema index are more difficult to control blood pressure [111].

### 2.6. Intervention Research

In a retrospective cohort study involving 118 patients with CKD, nutritional therapy was given to CKD patients with nutritional risk or malnutrition in the nutrition clinic. After 1 year of intervention, BMI and the percentage of body fat (BF%) measured by BIA were significantly higher than those before intervention, but there was no significant change in skeletal muscle mass (SMM) [112]. Another study of 148 elderly non-dialysis CKD4-5 subjects showed that the ketone analogue (KA) intervention group tended to preserve skeletal muscle and body fat mass (FM) compared with the control group, while non-KA users had a significant reduction in muscle mass (MM) and a significant increase in FM [113]. A study involving 240 non-diabetic patients with HD showed that, after 12 weeks of randomized administration of a fat-based, energy-intensive nutritional supplement, there was no significant improvement in nutritional status as measured by PhA compared with control [114]. Thirteen hemodialysis patients were longitudinally followed up for 12 months from conventional dialysis (3 × 4 h/week) to intensive nocturnal dialysis (3 × 8 h/week). It was found that extra-cellular water (ECW) measured by BIA was significantly lower than before. The phase angle (PhA) was significantly increased (6.2° versus 6.9°) [115]. In a randomized trial of a pedometer-based weekly activity goal intervention, participants in the intervention had a significantly greater increase in total body muscle mass (MM) of 0.7 kg/m^2^, decrease in FM (−4.3 kg) and decrease in BMI (−1.0 kg/m^2^) relative to controls after 6 months [116].

## 3. Conclusions

In this review, we first discussed the accuracy of BIA for body composition measurement in CKD patients, including its agreement with DEXA, the gold standard for body composition measurement, and its comparison with other anthropometric methods. It was concluded that the accuracy and advantages of the BIA method were still controversial. These studies suggested that BIA equations can be developed in patients with different types of CKD (pre-dialysis, HD, PD or KTR) or special conditions (edema or obesity) to improve the accuracy of BIA in the detection of body composition.

Next, this article separately described the research of BIA on nutritional management of CKD patients in pre-dialysis, hemodialysis, peritoneal dialysis and kidney transplantation, and summarized the relevant research conclusions from the indicators of lean body mass (muscle, skeletal muscle, body cell number), fat, phase angle and body water measured by body composition in each type of CKD patients. There were some consistent conclusions, which are summarized below. Firstly, lean tissue and fat: as eGFR decreased, LTI/MM/SMM/SMI/BCM decreased (pre-dialysis and KTR patients); lean mass index was positively correlated with protein intake within a certain range (pre-dialysis, HD and KTR patients). Lean mass index was positively correlated with grip strength and quality of life (pre-dialysis and HD patients). Low lean mass index was a risk factor for PEW/malnutrition (pre-dialysis, HD, PD and KTR patients). SMI was a good indicator to determine and predict sarcopenia (pre-dialysis, HD and KTR patients). The greater the percentage of visceral fat mass, the higher the risk of cardiovascular disease (HD and PD patients). Secondly, phase angle: The PhA of CKD patients was lower than that of healthy people, and the PhA of diabetic patients was lower than that of non-diabetic patients (pre-dialysis, HD and KTR patients). There was a negative correlation between inflammatory markers and PhA, and a positive correlation between albumin and PhA (pre-dialysis and KTR patients). PhA is a good predictor of PEW. The lower the PhA, the higher the risk of death (pre-dialysis, HD, PD and KTR patients). Thirdly, body water: CKD patients were prone to overhydration (OH) (pre-dialysis, HD, PD and KTR patients). Patients with higher levels of OH often had worse nutritional status (HD and PD patients). The higher the degree of OH, the higher the incidence of cardiovascular events (PD and KTR patients). OH was an independent predictor of mortality (pre-dialysis, HD, PD and KTR patients). The specific summary is shown in Table 1.

In addition, there were few studies on the effect evaluation of BIA in nutritional intervention, which may be the direction of application and research of BIA in CKD nutritional management in the future. In conclusion, the BIA method is of great practical value and clinical significance for the nutritional management of CKD patients.

## Figures and Tables

**Table 1:** Conclusions from the research of BIA on nutritional management in each type of CKD patients.

Index	Results	Pre-Dialysis CKD	HD	PD	KTR
Lean tissue and fat	GFR↓, LTI/MM/SMM/SMI/BCM↓	√			√
	Protein intake↓, Lean mass index↓	√	√		√
	Lean mass index↓, grip strength and quality of life↓	√	√		
	Lean mass index↓, PEW/malnutrition↑	√	√	√	√
	SMI↓, sarcopenia↑	√	√		√
	Visceral fat mass↑, cardiovascular disease↑		√	√	
Phase angle	PhA↓(CKD & Diabetes)	√	√		√
	Inflammatory markers↑, PhA↓	√			√
	Albumin↓, PhA↓	√			√
	PhA↓, PEW↑	√	√	√	√
	PhA↓, risk of death↑	√	√	√	√
Body water	Overhydration (OH)↑(CKD)	√	√	√	√
	OH↑, nutritional status↓		√	√	
	OH↑, cardiovascular event↑			√	√
	OH↑, mortality↑	√	√	√	√

CKD, chronic kidney disease; HD, hemodialysis; PD, peritoneal dialysis; KTR, kidney transplant recipients; GFR, glomerular filtration rate; LTI, lean tissue index; MM, muscle mass; SMM, skeletal muscle mass; SMI, skeletal muscle mass index; BCM, body cell mass; PEW, protein-energy wasting; PhA, phase angle. “↓” represents a lower value or a lower risk of occurrence or a worse state, “↑”represents a higher value or a higher risk of occurrence or a better state. “√” represents that the results have been confirmed by related studies in the corresponding stage of chronic kidney disease. The full meaning needs to match the content of the text.

## Data Availability

Not applicable.

## References

[1] Lemos T., Gallagher D. (2017). Current body composition measurement techniques. Curr. Opin. Endocrinol. Diabetes Obes..

[2] Kuriyan R. (2018). Body composition techniques. Indian J. Med. Res..

[3] Fosbol M.O., Zerahn B. (2015). Contemporary methods of body composition measurement. Clin. Physiol. Funct. Imaging.

[4] Lukaski H.C., Johnson P.E., Bolonchuk W.W., Lykken G.I. (1985). Assessment of fat-free mass using bioelectrical impedance measurements of the human body. Am. J. Clin. Nutr..

[5] Earthman C.P. (2015). Body Composition Tools for Assessment of Adult Malnutrition at the Bedside: A Tutorial on Research Considerations and Clinical Applications. JPEN J. Parenter. Enter. Nutr..

[6] Ward L.C. (2019). Bioelectrical impedance analysis for body composition assessment: Reflections on accuracy, clinical utility, and standardisation. Eur. J. Clin. Nutr..

[7] Lee S.W., Ngoh C.L.Y., Chua H.R., Haroon S., Wong W.K., Lee E.J., Lau T.W., Sethi S., Teo B.W. (2019). Evaluation of different bioimpedance methods for assessing body composition in Asian non-dialysis chronic kidney disease patients. Kidney Res. Clin. Pract..

[8] Broers N.J.H., Canaud B., Dekker M.J.E., van der Sande F.M., Stuard S., Wabel P., Kooman J.P. (2020). Three compartment bioimpedance spectroscopy in the nutritional assessment and the outcome of patients with advanced or end stage kidney disease: What have we learned so far?. Hemodial. Int. Int. Symp. Home Hemodial..

[9] Ikizler T.A., Burrowes J.D., Byham-Gray L.D., Campbell K.L., Carrero J.J., Chan W., Fouque D., Friedman A.N., Ghaddar S., Goldstein-Fuchs D.J. (2020). KDOQI Clinical Practice Guideline for Nutrition in CKD: 2020 Update. Am. J. Kidney Dis. Off. J. Natl. Kidney Found..

[10] Ekramzadeh M., Santoro D., Kopple J.D. (2022). The Effect of Nutrition and Exercise on Body Composition, Exercise Capacity, and Physical Functioning in Advanced CKD Patients. Nutrients.

[11] Marra M., Sammarco R., De Lorenzo A., Iellamo F., Siervo M., Pietrobelli A., Donini L.M., Santarpia L., Cataldi M., Pasanisi F. (2019). Assessment of Body Composition in Health and Disease Using Bioelectrical Impedance Analysis (BIA) and Dual Energy X-Ray Absorptiometry (DXA): A Critical Overview. Contrast Media Mol. Imaging.

[12] Furstenberg A., Davenport A. (2011). Comparison of multifrequency bioelectrical impedance analysis and dual-energy X-ray absorptiometry assessments in outpatient hemodialysis patients. Am. J. Kidney Dis. Off. J. Natl. Kidney Found..

[13] Furstenberg A., Davenport A. (2011). Assessment of body composition in peritoneal dialysis patients using bioelectrical impedance and dual-energy x-ray absorptiometry. Am. J. Nephrol..

[14] Chertow G.M., Lowrie E.G., Wilmore D.W., Gonzalez J., Lew N.L., Ling J., Leboff M.S., Gottlieb M.N., Huang W., Zebrowski B. (1995). Nutritional assessment with bioelectrical impedance analysis in maintenance hemodialysis patients. J. Am. Soc. Nephrol. JASN.

[15] Pelle G., Branche I., Kossari N., Tricot L., Delahousse M., Dreyfus J.F. (2013). Is 3-compartment bioimpedance spectroscopy useful to assess body composition in renal transplant patients?. J. Ren. Nutr..

[16] Bhandari R., Teh J.B., He T., Peng K., Iukuridze A., Atencio L., Nakamura R., Mostoufi-Moab S., McCormack S., Lee K. (2022). Association Between Body Composition and Development of Glucose Intolerance after Allogeneic Hematopoietic Cell Transplantation. Cancer Epidemiol. Biomark. Prev..

[17] Dou Y., Li A., Liu G., Wang P., Zhang B. (2023). Comparison of bioimpedance equations and dual-energy X-ray for assessment of fat free mass in a Chinese dialysis population. Ren. Fail..

[18] Eyre S., Bosaeus I., Jensen G., Saeed A. (2022). Using Bioimpedance Spectroscopy for Diagnosis of Malnutrition in Chronic Kidney Disease Stage 5-Is It Useful?. J. Ren. Nutr..

[19] Barreto Silva M.I., Menna Barreto A.P.M., Pontes K., Costa M.S.D., Rosina K.T.C., Souza E., Bregman R., Prado C.M., Klein M. (2021). Accuracy of surrogate methods to estimate skeletal muscle mass in non-dialysis dependent patients with chronic kidney disease and in kidney transplant recipients. Clin. Nutr..

[20] De Abreu A.M., Wilvert L.C., Wazlawik E. (2020). Comparison of Body Mass Index, Skinfold Thickness, and Bioelectrical Impedance Analysis With Dual-Energy X-Ray Absorptiometry in Hemodialysis Patients. Nutr. Clin. Pract..

[21] Bross R., Chandramohan G., Kovesdy C.P., Oreopoulos A., Noori N., Golden S., Benner D., Kopple J.D., Kalantar-Zadeh K. (2010). Comparing body composition assessment tests in long-term hemodialysis patients. Am. J. Kidney Dis..

[22] Rigalleau V., Lasseur C., Chauveau P., Barthes N., Raffaitin C., Combe C., Perlemoine C., Baillet-Blanco L., Gin H. (2004). Body composition in diabetic subjects with chronic kidney disease: Interest of bio-impedance analysis, and anthropometry. Ann. Nutr. Metab..

[23] Silva M.I., Vale B.S., Lemos C.C., Torres M.R., Bregman R. (2013). Body adiposity index assess body fat with high accuracy in nondialyzed chronic kidney disease patients. Obesity.

[24] Ravindranath J., Pillai P.P., Parameswaran S., Kamalanathan S.K., Pal G.K. (2016). Body Fat Analysis in Predialysis Chronic Kidney Disease: Multifrequency Bioimpedance Assay and Anthropometry Compared With Dual-Energy X-Ray Absorptiometry. J. Ren. Nutr..

[25] Kamimura M.A., Avesani C.M., Cendoroglo M., Canziani M.E., Draibe S.A., Cuppari L. (2003). Comparison of skinfold thicknesses and bioelectrical impedance analysis with dual-energy X-ray absorptiometry for the assessment of body fat in patients on long-term haemodialysis therapy. Nephrol. Dial. Transplant..

[26] Bellafronte N.T., Diani L.M., Vega-Piris L., Cuadrado G.B., Chiarello P.G. (2021). Comparison between dual-energy x-ray absorptiometry and bioelectrical impedance for body composition measurements in adults with chronic kidney disease: A cross-sectional, longitudinal, multi-treatment analysis. Nutrition.

[27] Melo D.A., Hortegal E.V.F., Guimaraes A., Franca A., Alves J., Santos E.M.D., Silva T., Silva J., Nunes L.C.R., Carvalho S.C.R. (2021). Sum of skinfolds measurement can be used in the estimation of total body fat in patients with chronic kidney disease undergoing hemodialysis. Nutr. Hosp..

[28] Ruperto M., Barril G. (2022). Nutritional Status, Body Composition, and Inflammation Profile in Older Patients with Advanced Chronic Kidney Disease Stage 4-5: A Case-Control Study. Nutrients.

[29] Kittiskulnam P., Nitesnoppakul M., Metta K., Suteparuk S., Praditpornsilpa K., Eiam-Ong S. (2021). Alterations of body composition patterns in pre-dialysis chronic kidney disease patients. Int. Urol. Nephrol..

[30] Liu L., Wang L., Wang X., Xiong M., Cao H., Jiang L., Yang J. (2022). Serum PTH Associated with Malnutrition Determined by Bioelectrical Impedance Technology in Chronic Kidney Disease Patients. Int. J. Endocrinol..

[31] Barril G., Nogueira A., Ruperto Lopez M., Castro Y., Sanchez-Tomero J.A. (2018). Influence of dietary protein intake on body composition in chronic kidney disease patients in stages 3-5: A cross-sectional study. Nefrologia.

[32] Jiang K., Singh Maharjan S.R., Slee A., Davenport A. (2021). Differences between anthropometric and bioimpedance measurements of muscle mass in the arm and hand grip and pinch strength in patients with chronic kidney disease. Clin. Nutr..

[33] Yongsiri S., Thammakumpee J., Prongnamchai S., Dinchuthai P., Chueansuwan R., Tangjaturonrasme S., Chaivanit P. (2014). The association between bioimpedance analysis and quality of life in pre-dialysis stage 5 chronic kidney disease, hemodialysis and peritoneal dialysis patients. J. Med. Assoc. Thail..

[34] Perez-Torres A., Gonzalez Garcia M.E., San Jose-Valiente B., Bajo Rubio M.A., Celadilla Diez O., Lopez-Sobaler A.M., Selgas R. (2018). Protein-energy wasting syndrome in advanced chronic kidney disease: Prevalence and specific clinical characteristics. Nefrologia.

[35] Bellafronte N.T., Sizoto G.R., Vega-Piris L., Chiarello P.G., Cuadrado G.B. (2020). Bed-side measures for diagnosis of low muscle mass, sarcopenia, obesity, and sarcopenic obesity in patients with chronic kidney disease under non-dialysis-dependent, dialysis dependent and kidney transplant therapy. PLoS ONE.

[36] Yu Z., Grams M.E., Ndumele C.E., Wagenknecht L., Boerwinkle E., North K.E., Rebholz C.M., Giovannucci E.L., Coresh J. (2021). Association Between Midlife Obesity and Kidney Function Trajectories: The Atherosclerosis Risk in Communities (ARIC) Study. Am. J. Kidney Dis..

[37] Vettoretti S., Caldiroli L., Armelloni S., Ferrari C., Cesari M., Messa P. (2019). Sarcopenia is Associated with Malnutrition but Not with Systemic Inflammation in Older Persons with Advanced CKD. Nutrients.

[38] Bhat P.R., Urooj A., Nalloor S. (2022). Changes in body composition in relation to estimated glomerular filtration rate and physical activity in predialysis chronic kidney disease. Chronic Dis. Transl. Med..

[39] Pereira R.A., Cordeiro A.C., Avesani C.M., Carrero J.J., Lindholm B., Amparo F.C., Amodeo C., Cuppari L., Kamimura M.A. (2015). Sarcopenia in chronic kidney disease on conservative therapy: Prevalence and association with mortality. Nephrol. Dial. Transplant..

[40] Han B.G., Lee J.Y., Kim J.S., Yang J.W. (2019). Decreased Bioimpedance Phase Angle in Patients with Diabetic Chronic Kidney Disease Stage 5. Nutrients.

[41] Bellizzi V., Scalfi L., Terracciano V., De Nicola L., Minutolo R., Marra M., Guida B., Cianciaruso B., Conte G., Di Iorio B.R. (2006). Early changes in bioelectrical estimates of body composition in chronic kidney disease. J. Am. Soc. Nephrol. JASN.

[42] Seo Y.K., Lee H., Kim H., Kim T.Y., Ryu H., Ju D.L., Jang M., Oh K.H., Ahn C., Han S.N. (2020). Foods contributing to nutrients intake and assessment of nutritional status in pre-dialysis patients: A cross-sectional study. BMC Nephrol..

[43] Han B.G., Lee J.Y., Kim J.S., Yang J.W. (2018). Clinical Significance of Phase Angle in Non-Dialysis CKD Stage 5 and Peritoneal Dialysis Patients. Nutrients.

[44] Barril G., Nogueira A., Alvarez-Garcia G., Nunez A., Sanchez-Gonzalez C., Ruperto M. (2022). Nutritional Predictors of Mortality after 10 Years of Follow-Up in Patients with Chronic Kidney Disease at a Multidisciplinary Unit of Advanced Chronic Kidney Disease. Nutrients.

[45] Wang K., Zelnick L.R., Chertow G.M., Himmelfarb J., Bansal N. (2021). Body Composition Changes Following Dialysis Initiation and Cardiovascular and Mortality Outcomes in CRIC (Chronic Renal Insufficiency Cohort): A Bioimpedance Analysis Substudy. Kidney Med..

[46] Hassan M.O., Duarte R., Dix-Peek T., Vachiat A., Dickens C., Grinter S., Naidoo S., Manga P., Naicker S. (2016). Volume overload and its risk factors in South African chronic kidney disease patients: An appraisal of bioimpedance spectroscopy and inferior vena cava measurements. Clin. Nephrol..

[47] Dumler F., Kilates C. (2005). Prospective nutritional surveillance using bioelectrical impedance in chronic kidney disease patients. J. Ren. Nutr..

[48] Ohashi Y., Otani T., Tai R., Tanaka Y., Sakai K., Aikawa A. (2013). Assessment of body composition using dry mass index and ratio of total body water to estimated volume based on bioelectrical impedance analysis in chronic kidney disease patients. J. Ren. Nutr..

[49] Chua H.R., Xiang L., Chow P.Y., Xu H., Shen L., Lee E., Teo B.W. (2012). Quantifying acute changes in volume and nutritional status during haemodialysis using bioimpedance analysis. Nephrology.

[50] Aatif T., Hassani K., Alayoud A., Maoujoud O., Ahid S., Benyahia M., Oualim Z. (2013). Parameters to assess nutritional status in a Moroccan hemodialysis cohort. Arab J. Nephrol. Transplant..

[51] Beberashvili I., Sinuani I., Azar A., Yasur H., Feldman L., Efrati S., Averbukh Z., Weissgarten J. (2009). Nutritional and inflammatory status of hemodialysis patients in relation to their body mass index. J. Ren. Nutr..

[52] Torun D., Micozkadioglu H., Torun N., Ozelsancak R., Sezer S., Adam F.U., Ozdemir F.N., Haberal M. (2007). Increased body mass index is not a reliable marker of good nutrition in hemodialysis patients. Ren. Fail..

[53] Garagarza C., Flores A.L., Valente A. (2018). Influence of Body Composition and Nutrition Parameters in Handgrip Strength: Are There Differences by Sex in Hemodialysis Patients?. Nutr. Clin. Pract..

[54] Song H.C., Shin J., Hwang J.H., Kim S.H. (2023). Utility of the Global Leadership Initiative on Malnutrition criteria for the nutritional assessment of patients with end-stage renal disease receiving chronic hemodialysis. J. Hum. Nutr. Diet..

[55] Vannini F.D., Antunes A.A., Caramori J.C., Martin L.C., Barretti P. (2009). Associations between nutritional markers and inflammation in hemodialysis patients. Int. Urol. Nephrol..

[56] Mayrink Ivo J.F., Sugizaki C.S.A., Souza Freitas A.T.V., Costa N.A., Peixoto M. (2023). Age, hemodialysis time, gait speed, but not mortality, are associated with muscle quality index in end-stage renal disease. Exp. Gerontol..

[57] Segall L., Mardare N.G., Ungureanu S., Busuioc M., Nistor I., Enache R., Marian S., Covic A. (2009). Nutritional status evaluation and survival in haemodialysis patients in one centre from Romania. Nephrol. Dial. Transplant..

[58] Segall L., Covic A., Mardare N., Ungureanu S., Marian S., Busuioc M., Nistor I., Enache R., Veisa G., Covic M. (2008). Nutritional status evaluation in maintenance hemodialysis patients. Rev. Med. Chir. A Soc. Med. Nat. Din Iasi.

[59] Rymarz A., Bartoszewicz Z., Szamotulska K., Niemczyk S. (2016). The Associations Between Body Cell Mass and Nutritional and Inflammatory Markers in Patients With Chronic Kidney Disease and in Subjects Without Kidney Disease. J. Ren. Nutr..

[60] Da J., Long Y., Li Q., Yang X., Yuan J., Zha Y. (2021). Resting metabolic rate and its adjustments as predictors of risk protein-energy wasting in hemodialysis patients. Biosci. Rep..

[61] Kamimura M.A., Draibe S.A., Dalboni M.A., Cendoroglo M., Avesani C.M., Manfredi S.R., Canziani M.E., Cuppari L. (2007). Serum and cellular interleukin-6 in haemodialysis patients: Relationship with energy expenditure. Nephrol. Dial. Transplant..

[62] Skouroliakou M., Stathopoulou M., Koulouri A., Giannopoulou I., Stamatiades D., Stathakis C. (2009). Determinants of resting energy expenditure in hemodialysis patients, and comparison with healthy subjects. J. Ren. Nutr..

[63] Marcelli D., Brand K., Ponce P., Milkowski A., Marelli C., Ok E., Merello Godino J.I., Gurevich K., Jirka T., Rosenberger J. (2016). Longitudinal Changes in Body Composition in Patients After Initiation of Hemodialysis Therapy: Results From an International Cohort. J. Ren. Nutr..

[64] Duong T.V., Wu P.Y., Wong T.C., Chen H.H., Chen T.H., Hsu Y.H., Peng S.J., Kuo K.L., Liu H.C., Lin E.T. (2019). Mid-arm circumference, body fat, nutritional and inflammatory biomarkers, blood glucose, dialysis adequacy influence all-cause mortality in hemodialysis patients: A prospective cohort study. Medicine.

[65] Delgado C., Chertow G.M., Kaysen G.A., Dalrymple L.S., Kornak J., Grimes B., Johansen K.L. (2017). Associations of Body Mass Index and Body Fat With Markers of Inflammation and Nutrition Among Patients Receiving Hemodialysis. Am. J. Kidney Dis..

[66] Arslan N. (2023). Association of cardiometabolic risks with body composition in hemodialysis patients. Eur. Rev. Med. Pharmacol. Sci..

[67] Erdogan E., Tutal E., Uyar M.E., Bal Z., Demirci B.G., Sayin B., Sezer S. (2013). Reliability of bioelectrical impedance analysis in the evaluation of the nutritional status of hemodialysis patients—A comparison with Mini Nutritional Assessment. Transplant. Proc..

[68] Valente A., Caetano C., Oliveira T., Garagarza C. (2019). Evaluating haemodialysis patient’s nutritional status: Body mass index or body cell mass index?. Nephrology.

[69] Arias-Guillen M., Perez E., Herrera P., Romano B., Ojeda R., Vera M., Rios J., Fontsere N., Maduell F. (2018). Bioimpedance Spectroscopy as a Practical Tool for the Early Detection and Prevention of Protein-Energy Wasting in Hemodialysis Patients. J. Ren. Nutr..

[70] Macedo C., Amaral T.F., Rodrigues J., Santin F., Avesani C.M. (2021). Malnutrition and Sarcopenia Combined Increases the Risk for Mortality in Older Adults on Hemodialysis. Front. Nutr..

[71] Tan R.S., Liang D.H., Liu Y., Zhong X.S., Zhang D.S., Ma J. (2019). Bioelectrical Impedance Analysis-Derived Phase Angle Predicts Protein-Energy Wasting in Maintenance Hemodialysis Patients. J. Ren. Nutr..

[72] Huang Y., Ge Y., Li F., Zhang C., Zhang Z., Xu N., Wang R., Wu S., Geng X., Quan Y. (2022). Elucidating the relationship between nutrition indices and coronary artery calcification in patients undergoing maintenance hemodialysis. Ther. Apher. Dial..

[73] Leal Escobar G., Osuna Padilla I.A., Cano Escobar K.B., Moguel Gonzalez B., Perez Grovas H.A., Ruiz Ubaldo S. (2019). Phase angle and mid arm circumference as predictors of protein energy wasting in renal replacement therapy patients. Nutr. Hosp..

[74] Topete-Reyes J.F., Lopez-Lozano C.A., Lopez-Baez S.L., Barbarin-Vazquez A.V., Cervantes-Villalobos M.L., Navarro-Rodriguez J., Parra-Michel R., Pazarin-Villasenor H.L., Meza-Guillen D., Torres-Tamayo M. (2019). Determinacion del estado nutricional mediante el angulo de fase en pacientes en hemodialisis. Gac. Med. Mex..

[75] Shin J.H., Kim C.R., Park K.H., Hwang J.H., Kim S.H. (2017). Predicting clinical outcomes using phase angle as assessed by bioelectrical impedance analysis in maintenance hemodialysis patients. Nutrition.

[76] Pupim L.B., Caglar K., Hakim R.M., Shyr Y., Ikizler T.A. (2004). Uremic malnutrition is a predictor of death independent of inflammatory status. Kidney Int..

[77] Beberashvili I., Azar A., Sinuani I., Shapiro G., Feldman L., Stav K., Sandbank J., Averbukh Z. (2014). Bioimpedance phase angle predicts muscle function, quality of life and clinical outcome in maintenance hemodialysis patients. Eur. J. Clin. Nutr..

[78] Tabinor M., Elphick E., Dudson M., Kwok C.S., Lambie M., Davies S.J. (2018). Bioimpedance-defined overhydration predicts survival in end stage kidney failure (ESKF): Systematic review and subgroup meta-analysis. Sci. Rep..

[79] Garagarza C., Joao-Matias P., Sousa-Guerreiro C., Amaral T., Aires I., Ferreira C., Jorge C., Gil C., Ferreira A. (2013). Nutritional status and overhydration: Can bioimpedance spectroscopy be useful in haemodialysis patients?. Nefrologia.

[80] Sukackiene D., Laucyte-Cibulskiene A., Vickiene A., Rimsevicius L., Miglinas M. (2020). Risk stratification for patients awaiting kidney transplantation: Role of bioimpedance derived edema index and nutrition status. Clin. Nutr..

[81] Ruperto M., Barril G. (2022). The Extracellular Mass to Body Cell Mass Ratio as a Predictor of Mortality Risk in Hemodialysis Patients. Nutrients.

[82] Garcia-Lopes M.G., Agliussi R.G., Avesani C.M., Manfredi S.R., Bazanelli A.P., Kamimura M.A., Draibe S.A., Cuppari L. (2008). Nutritional status and body composition after 6 months of patients switching from continuous ambulatorial peritoneal dialysis to automated peritoneal dialysis. Braz. J. Med. Biol. Res..

[83] Kanazawa Y., Nakao T., Matsumoto H., Okada T., Hidaka H., Yoshino M., Shino T., Nagaoka Y., Takeguchi F., Iwasawa H. (2001). Serial changes in body composition in patients with chronic renal failure on peritoneal dialysis. Nihon Jinzo Gakkai Shi.

[84] As’habi A., Najafi I., Tabibi H., Hedayati M. (2018). Prevalence of Sarcopenia and Dynapenia and Their Determinants in Iranian Peritoneal Dialysis Patients. Iran. J. Kidney Dis..

[85] Fan J., Ye H., Zhang X., Cao P., Guo Q., Mao H., Yu X., Yang X. (2019). Association of Lean Body Mass Index and Peritoneal Protein Clearance in Peritoneal Dialysis Patients. Kidney Blood Press. Res..

[86] Tabibi H., As’habi A., Najafi I., Hedayati M. (2018). Prevalence of dynapenic obesity and sarcopenic obesity and their associations with cardiovascular disease risk factors in peritoneal dialysis patients. Kidney Res. Clin. Pract..

[87] Kim C., Kim J.K., Lee H.S., Kim S.G., Song Y.R. (2021). Longitudinal changes in body composition are associated with all-cause mortality in patients on peritoneal dialysis. Clin. Nutr..

[88] Lu Q., Cheng L.T., Wang T., Wan J., Liao L.L., Zeng J., Qin C., Li K.J. (2008). Visceral fat, arterial stiffness, and endothelial function in peritoneal dialysis patients. J. Ren. Nutr..

[89] Reichel G. (1974). Results of continous therapy with atrovent in diurnal and long-term administration. Wien. Med. Wochenschrift. Suppl..

[90] Cheng L.T., Tang W., Wang T. (2005). Strong association between volume status and nutritional status in peritoneal dialysis patients. Am. J. Kidney Dis..

[91] Guo Q., Lin J., Li J., Yi C., Mao H., Yang X., Yu X. (2015). The Effect of Fluid Overload on Clinical Outcome in Southern Chinese Patients Undergoing Continuous Ambulatory Peritoneal Dialysis. Perit. Dial. Int. J. Int. Soc. Perit. Dial..

[92] Demirci M.S., Demirci C., Ozdogan O., Kircelli F., Akcicek F., Basci A., Ok E., Ozkahya M. (2011). Relations between malnutrition-inflammation-atherosclerosis and volume status. The usefulness of bioimpedance analysis in peritoneal dialysis patients. Nephrol. Dial. Transplant..

[93] Avram M.M., Mittman N., Fein P.A., Agahiu S., Hartman W., Chattopadhyay N., Matza B. (2012). Dialysis vintage, body composition, and survival in peritoneal dialysis patients. Advances in Peritoneal dialysis. Conference on Peritoneal Dialysis.

[94] Jones C.H., Newstead C.G. (2004). The ratio of extracellular fluid to total body water and technique survival in peritoneal dialysis patients. Perit. Dial. Int. J. Int. Soc. Perit. Dial..

[95] Avram M.M., Fein P.A., Borawski C., Chattopadhyay J., Matza B. (2010). Extracellular mass/body cell mass ratio is an independent predictor of survival in peritoneal dialysis patients. Kidney Int. Suppl..

[96] O’Lone E.L., Visser A., Finney H., Fan S.L. (2014). Clinical significance of multi-frequency bioimpedance spectroscopy in peritoneal dialysis patients: Independent predictor of patient survival. Nephrol. Dial. Transplant..

[97] Tutal E., Sezer S., Uyar M.E., Bal Z., Demirci B.G., Acar F.N. (2013). Evaluation of nutritional status in renal transplant recipients in accordance with changes in graft function. Transplant. Proc..

[98] Nanmoku K., Kawabata N., Kinoshita Y., Shinzato T., Kubo T., Shimizu T., Yagisawa T. (2020). Deterioration of presarcopenia and its risk factors following kidney transplantation. Clin. Exp. Nephrol..

[99] Sukackiene D., Rimsevicius L., Miglinas M. (2022). Standardized Phase Angle for Predicting Nutritional Status of Hemodialysis Patients in the Early Period After Deceased Donor Kidney Transplantation. Front. Nutr..

[100] Kosoku A., Iwai T., Ishihara T., Kabei K., Nishide S., Maeda K., Hanayama Y., Ishimura E., Uchida J. (2022). Influence of protein intake on the changes in skeletal muscle mass after kidney transplantation. Clin. Nutr..

[101] Heleniak Z., Illersperger S., Malgorzewicz S., Debska-Slizien A., Budde K., Halleck F. (2022). Arterial Stiffness as a Cardiovascular Risk Factor After Successful Kidney Transplantation in Diabetic and Nondiabetic Patients. Transplant. Proc..

[102] Saxena A., Sharma R.K., Gupta A. (2016). Graft function and nutritional parameters in stable postrenal transplant patients. Saudi J. Kidney Dis. Transplant..

[103] Boslooper-Meulenbelt K., van Vliet I.M.Y., Gomes-Neto A.W., de Jong M.F.C., Bakker S.J.L., Jager-Wittenaar H., Navis G.J. (2021). Malnutrition according to GLIM criteria in stable renal transplant recipients: Reduced muscle mass as predominant phenotypic criterion. Clin. Nutr..

[104] Dos Reis A.S., Limirio L.S., Santos H.O., de Oliveira E.P. (2021). Intake of polyunsaturated fatty acids and omega-3 are protective factors for sarcopenia in kidney transplant patients. Nutrition.

[105] Wong H.S., Boey L.M., Morad Z. (2004). Body composition by bioelectrical impedance analysis in renal transplant recipients. Transplant. Proc..

[106] Kosoku A., Uchida J., Nishide S., Kabei K., Shimada H., Iwai T., Maeda K., Hanayama Y., Ishihara T., Naganuma T. (2020). Association of sarcopenia with phase angle and body mass index in kidney transplant recipients. Sci. Rep..

[107] Kaya E., Bakir A., Koseoglu Y.K., Velidedeoglu M., Trabulus S., Seyahi N. (2019). Association of Nutritional Assessment by Phase Angle With Mortality in Kidney Transplant Patients in an 8-Year Follow-Up. Prog. Transplant..

[108] Abenoza P., Manivel J.C., Wick M.R., Hagen K., Dehner L.P. (1987). Hepatoblastoma: An immunohistochemical and ultrastructural study. Hum. Pathol..

[109] Coroas A.S., Oliveira J.G., Sampaio S., Borges C., Tavares I., Pestana M., Almeida M.D. (2006). Body composition assessed by impedance changes very early with declining renal graft function. Nephron. Physiol..

[110] Nishimura N., Hori S., Tomizawa M., Yoneda T., Nakai Y., Miyake M., Torimoto K., Tanaka N., Fujimoto K. (2022). Relevance of the perioperative edema index measured by bioelectrical impedance analysis for prediction of cardiovascular disease in living-donor kidney transplantation. Int. J. Urol..

[111] Saxena A., Sharma R.K. (2014). Hypertension in post-renal transplant patients: Pilot study. Saudi J. Kidney Dis. Transplant..

[112] Saka B., Bektas M., Bakkaloglu O.K., Amikishiyev S., Saribeyliler G., Tiryaki T.O., Ince B., Cakmak R., Buyukdemir S., Senturk B.O. (2022). Malnutrition treatment and follow-up in clinical nutrition outpatient clinics associated with increased muscle mass. Nutrition.

[113] Lin Y.L., Hou J.S., Wang C.H., Su C.Y., Liou H.H., Hsu B.G. (2021). Effects of ketoanalogues on skeletal muscle mass in patients with advanced chronic kidney disease: Real-world evidence. Nutrition.

[114] Yang Y., Qin X., Chen J., Wang Q., Kong Y., Wan Q., Tao H., Liu A., Li Y., Lin Z. (2021). The Effects of Oral Energy-Dense Supplements on Nutritional Status in Nondiabetic Maintenance Hemodialysis Patients: A Randomized Controlled Trial. Clin. J. Am. Soc. Nephrol. CJASN.

[115] David S., Kumpers P., Eisenbach G.M., Haller H., Kielstein J.T. (2009). Prospective evaluation of an in-centre conversion from conventional haemodialysis to an intensified nocturnal strategy. Nephrol. Dial. Transplant..

[116] Sheshadri A., Kittiskulnam P., Lai J.C., Johansen K.L. (2020). Effect of a pedometer-based walking intervention on body composition in patients with ESRD: A randomized controlled trial. BMC Nephrol..

